# Expression of Wnt Signaling Components during *Xenopus* Pronephros Development

**DOI:** 10.1371/journal.pone.0026533

**Published:** 2011-10-19

**Authors:** Bo Zhang, Uyen Tran, Oliver Wessely

**Affiliations:** 1 Lerner Research Institute/Cleveland Clinic, Department of Cell Biology, Cleveland, Ohio, United States of America; 2 Louisiana State University (LSU) Health Sciences Center, Department of Cell Biology & Anatomy, New Orleans, Louisiana, United States of America; National Cancer Institute, United States of America

## Abstract

**Background:**

The formation of the vertebrate kidney is tightly regulated and relies on multiple evolutionarily conserved inductive events. These are present in the complex metanephric kidney of higher vertebrates, but also in the more primitive pronephric kidney functional in the larval stages of amphibians and fish. Wnts have long been viewed as central in this process. Canonical β-Catenin-dependent Wnt signaling establishes kidney progenitors and non-canonical β-Catenin-independent Wnt signaling participate in the morphogenetic processes that form the highly sophisticated nephron structure. While some individual Wnt signaling components have been studied extensively in the kidney, the overall pathway has not yet been analyzed in depth.

**Methodology/Principal Findings:**

Here we report a detailed expression analysis of all Wnt ligands, receptors and several downstream Wnt effectors during pronephros development in *Xenopus laevis* using *in situ* hybridization. Out of 19 Wnt ligands, only three, *Wnt4*, *Wnt9a* and *Wnt11*, are specifically expressed in the pronephros. Others such as *Wnt8a* are present, but in a broader domain comprising adjacent tissues in addition to the kidney. The same paradigm is observed for the Wnt receptors and its downstream signaling components. *Fzd1*, *Fzd4*, *Fzd6*, *Fzd7*, *Fzd8* as well as *Celsr1* and *Prickle1* show distinct expression domains in the pronephric kidney, whereas the non-traditional Wnt receptors, *Ror2* and *Ryk*, as well as the majority of the effector molecules are rather ubiquitous. In addition to this spatial regulation, the timing of expression is also tightly regulated. In particular, non-canonical Wnt signaling seems to be restricted to later stages of pronephros development.

**Conclusion/Significance:**

Together these data suggest a complex cross talk between canonical and non-canonical Wnt signaling is required to establish a functional pronephric kidney.

## Introduction

In vertebrates the kidney is an essential organ required to excrete nitrogenous waste and maintain salt and water balance. Embryologically, the kidney is derived from the intermediate mesoderm and develops through three increasingly complex forms, the pronephros, the mesonephros and the metanephros. While the mesonephros is the adult kidney of fish and amphibians, the metanephros is found in higher vertebrates such as mammals and avians [1], [2], [3]; the pronephros, the most primitive kidney form is present in all vertebrates during embryonic development. It is often not functional and degenerates later on, but is of particular importance for larval stages of aquatic animals and is necessary to regulate their osmotic balance with the surrounding medium [2]. Even though the three kidney forms are morphologically quite distinct, they are all based on the same functional unit, the nephron. Molecular analyses have demonstrated that many aspects of nephron formation and patterning in the three kidney forms are evolutionarily conserved [4], [5], [6].

Wnt signaling is one of the most critical pathways in many aspects of development [7], [8], [9], [10], [11]. Wnt ligands bind to the Frizzled family of G protein-coupled receptor-like proteins. Upon binding they recruit the adaptor protein Disheveled (Dvl) and trigger a variety of downstream signaling events. Wnt signaling is subdivided into the canonical β-Catenin-dependent and the non-canonical β-Catenin-independent branch. Canonical Wnt signaling is characterized by the stabilization and subsequent nuclear transport of β-Catenin resulting in the activation of transcriptional responses. Non-canonical Wnt signaling is more diverse and includes several different signaling modes that regulate cell behavior. These include the phospholipase C-mediated increase of Ca^2+^, modification of the actin cytoskeleton by the small G proteins Rac and Rho and the regulation of planar cell polarity (PCP) by the PCP core proteins Prickle, Van Gogh-like (Vangl, also known as Strabismus) and Celsr. Importantly, Wnt signaling is no longer restricted to only Frizzled receptors, but non-Frizzled receptors like Ror2 and Ryk have emerged as well [11], [12].

The diversity of Wnt signaling is reflected by the fact that 19 different Wnts, 10 Frizzled receptors, three disheveled as well as multiple members of the PCP core proteins, Celsr, Vangl and Prickle have been identified in most vertebrates (Figures S1, S2, S3). Moreover, different Wnts can regulate different aspects at the same time. For example, in mouse kidney development Wnt9b maintains the mesenchymal progenitor cell population that gives rise to the individual nephrons [13], [14], while Wnt4 regulates the formation of the renal vesicle [15], [16]. Compared to the Wnt ligands, the receptors as well as the downstream regulators are much less studied. To embrace the complexity of the Wnt signaling pathway and understand the cross talk between the different branches (i.e. canonical and non-canonical), we decided to examine the expression of all Wnts, their receptors and several downstream signaling components using the *Xenopus* pronephros as a paradigm. Using *in situ* hybridization we identified pronephric kidney-specific expression for *Wnt4*, *Wnt9a*, *Wnt11*, *Fzd1*, *Fzd4*, *Fzd6*, *Fzd7*, *Fzd8* as well as the PCP core proteins *Celsr1* and *Prickle1*. Moreover, these expression domains were distinct not only in respect to their spatial domains, but also in their temporal aspect. This suggests that even in the rather primitive pronephros Wnt signaling has to be tightly controlled to assure proper organ growth.

## Results

### Expression of Wnt Ligands

**Figure 1:**
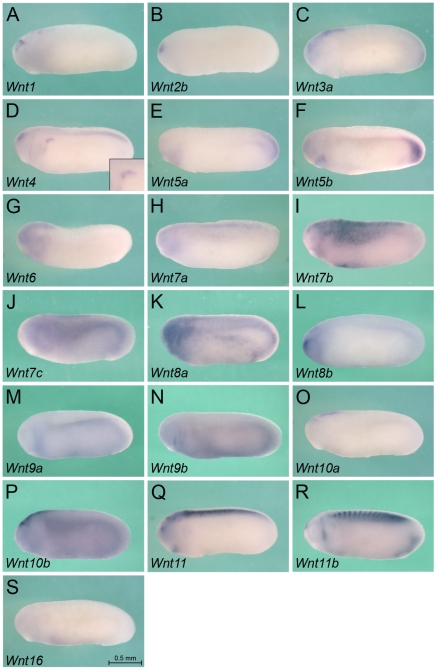
Expression of Wnt Ligands at Stage 25. Whole mount *in situ* hybridization of *Xenopus* embryos at stage 25 for *Wnt1* (A), *Wnt2b* (B), *Wnt3a* (C), *Wnt4* (D), *Wnt5a* (E), *Wnt5b* (F), *Wnt6* (G), *Wnt7a* (H), *Wnt7b* (I), *Wnt7c* (J), *Wnt8a* (K), *Wnt8b* (L), *Wnt9a* (M), *Wnt9b* (N), *Wnt10a* (O), *Wnt10b* (P), *Wnt11* (Q), *Wnt11b* (R) and *Wnt16* (S). Inset in D shows close-up of the pronephric anlage. All images are of the same magnification and the scale bar corresponds to 0.5 mm.

While Wnt signaling components have been previously analyzed in *Xenopus* (see below), their expression was not studied in respect to pronephros formation. Thus, we decided to systematically monitor the expression patterns of the entire Wnt family, its receptors and downstream signaling components by whole mount *in situ* hybridization (Figures 1-6) and subsequent sectioning (Figure 7). In *Xenopus*, 19 different Wnt molecules have been identified (Figure S1, [11]). Since pronephros development starts around stage 12.5 and the kidney becomes functional at stage 38 [5], [17], we focused on this interval. We examined multiple stages in between these time points, but the subsequent figures depict two stages: stage 25, when the pronephros has been specified, but mesenchymal-epithelial transition required for tubulogenesis has not yet commenced; stage 35, when the epithelial tubules have formed and the pronephros is patterned along the proximo-distal axis.

**Figure 2 pone-0026533-g002:**
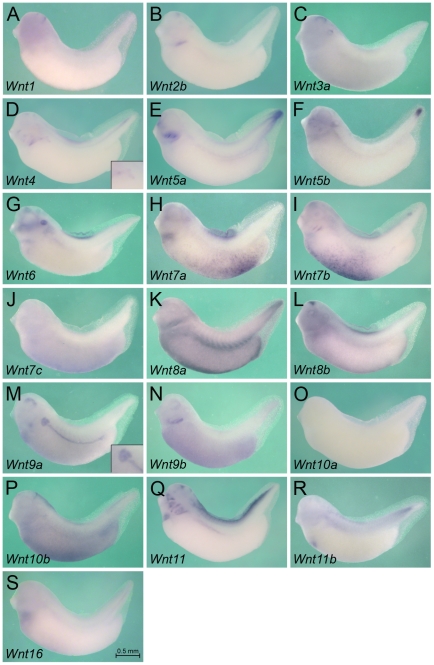
Expression of Wnt Ligands at Stage 35. Whole mount *in situ* hybridization of *Xenopus* embryos at stage 35 for *Wnt1* (A), *Wnt2b* (B), *Wnt3a* (C), *Wnt4* (D), *Wnt5a* (E), *Wnt5b* (F), *Wnt6* (G), *Wnt7a* (H), *Wnt7b* (I), *Wnt7c* (J), *Wnt8a* (K), *Wnt8b* (L), *Wnt9a* (M), *Wnt9b* (N), *Wnt10a* (O), *Wnt10b* (P), *Wnt11* (Q), *Wnt11b* (R) and *Wnt16* (S). Insets in D and M show close-ups of the pronephric tubular region. All images are of the same magnification and the scale bar corresponds to 0.5 mm.

**Figure 3 pone-0026533-g003:**
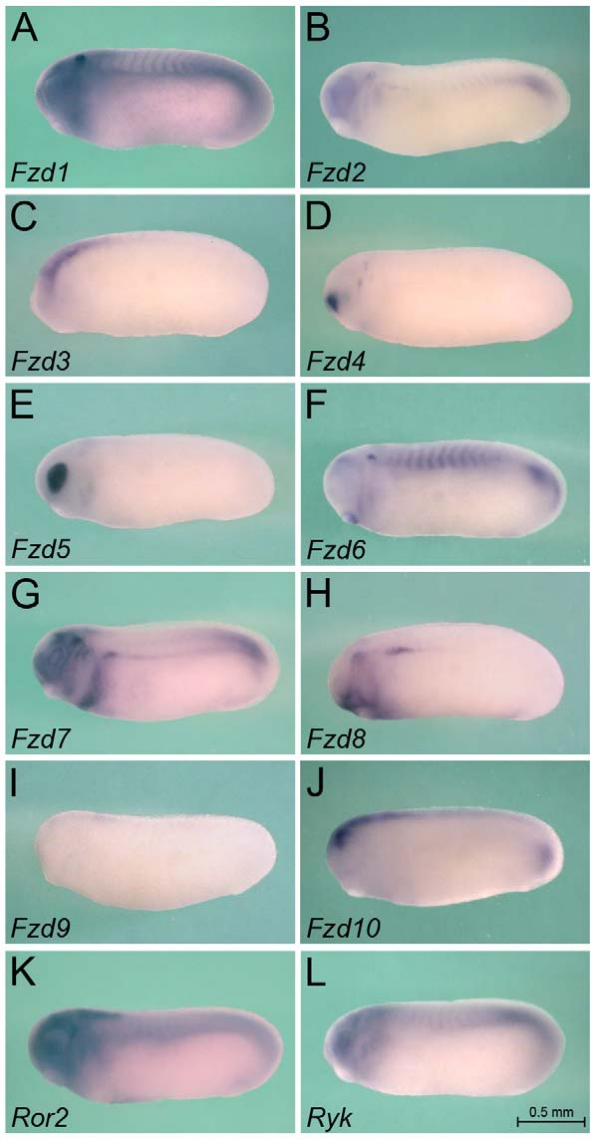
Expression of Wnt Receptors at Stage 25. Whole mount *in situ* hybridization of *Xenopus* embryos at stage 25 for *Fzd1* (A), *Fzd2* (B), *Fzd3* (C), *Fzd4* (D), *Fzd5* (E), *Fzd6* (F), *Fzd7* (G), *Fzd8* (H), *Fzd9* (I), *Fzd10* (J), *Ror2* (K) and *Ryk* (L). All images are of the same magnification and the scale bar corresponds to 0.5 mm.

**Figure 4 pone-0026533-g004:**
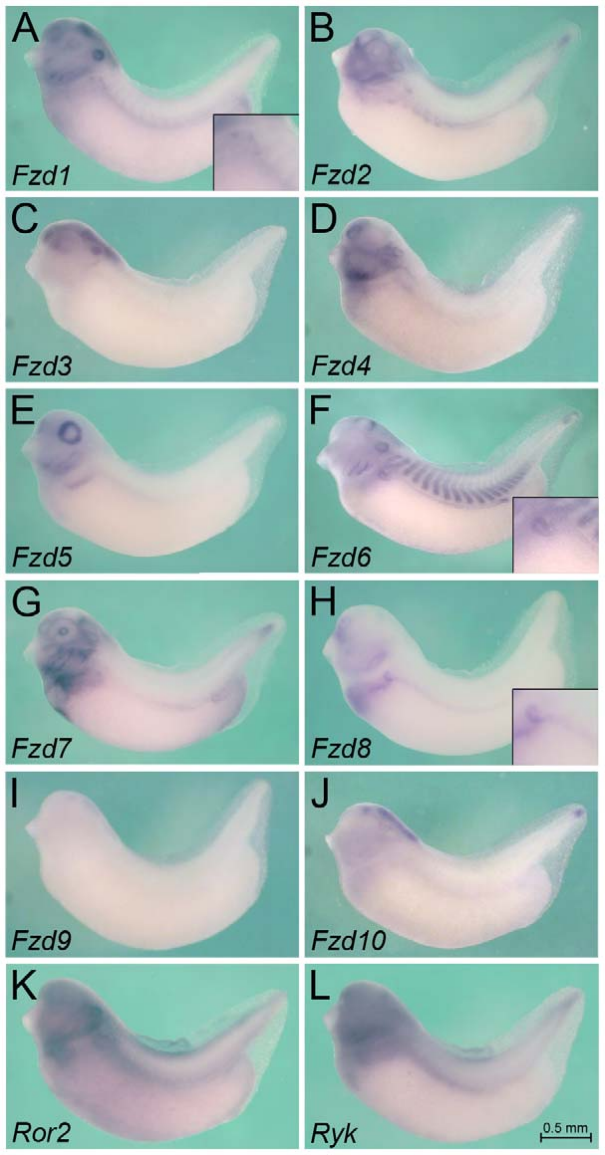
Expression of Wnt Receptors at Stage 35. Whole mount *in situ* hybridization of *Xenopus* embryos at stage 35 for *Fzd1* (A), *Fzd2* (B), *Fzd3* (C), *Fzd4* (D), *Fzd5* (E), *Fzd6* (F), *Fzd7* (G), *Fzd8* (H), *Fzd9* (I), *Fzd10* (J), *Ror2* (K) and *Ryk* (L). Inset in A, F and H show close-ups of the pronephric tubular region. All images are of the same magnification and the scale bar corresponds to 0.5 mm.

**Figure 5 pone-0026533-g005:**
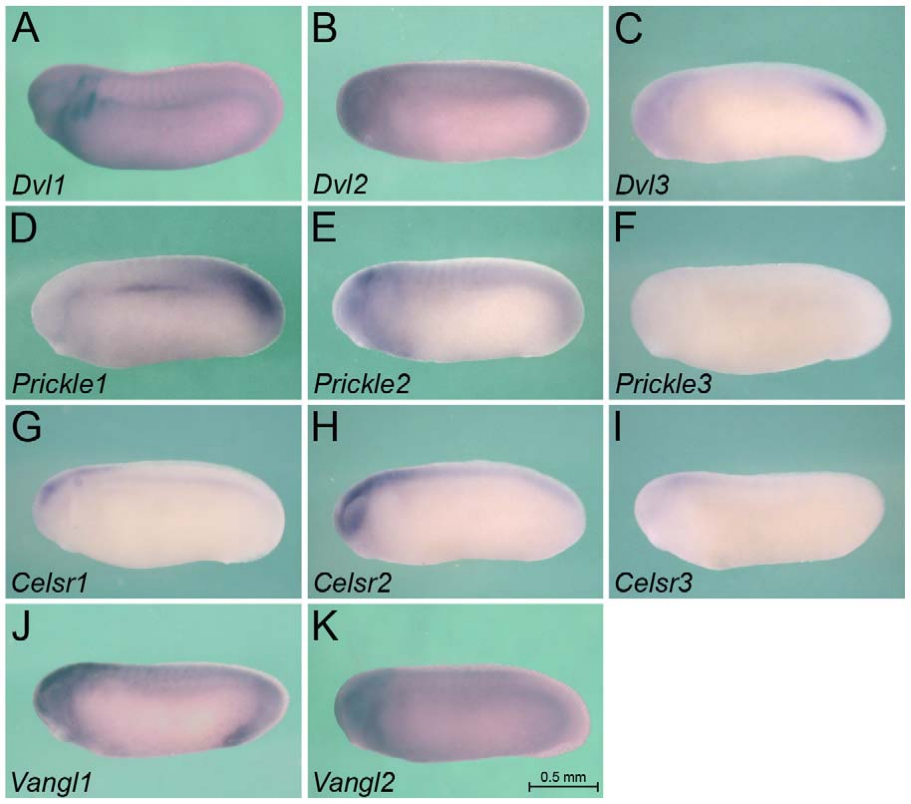
Expression of Wnt Signaling Intermediates at Stage 25. Whole mount *in situ* hybridization of *Xenopus* embryos at stage 25 for *Dvl1* (A), *Dvl2* (B), *Dvl3* (C), *Prickle1* (D), *Prickle2* (E), *Prickle3* (F), *Celsr1* (G), *Celsr2* (H), *Celsr3* (I), *Vangl1* (J) and *Vangl2* (K). All images are of the same magnification and the scale bar corresponds to 0.5 mm.

**Figure 6:**
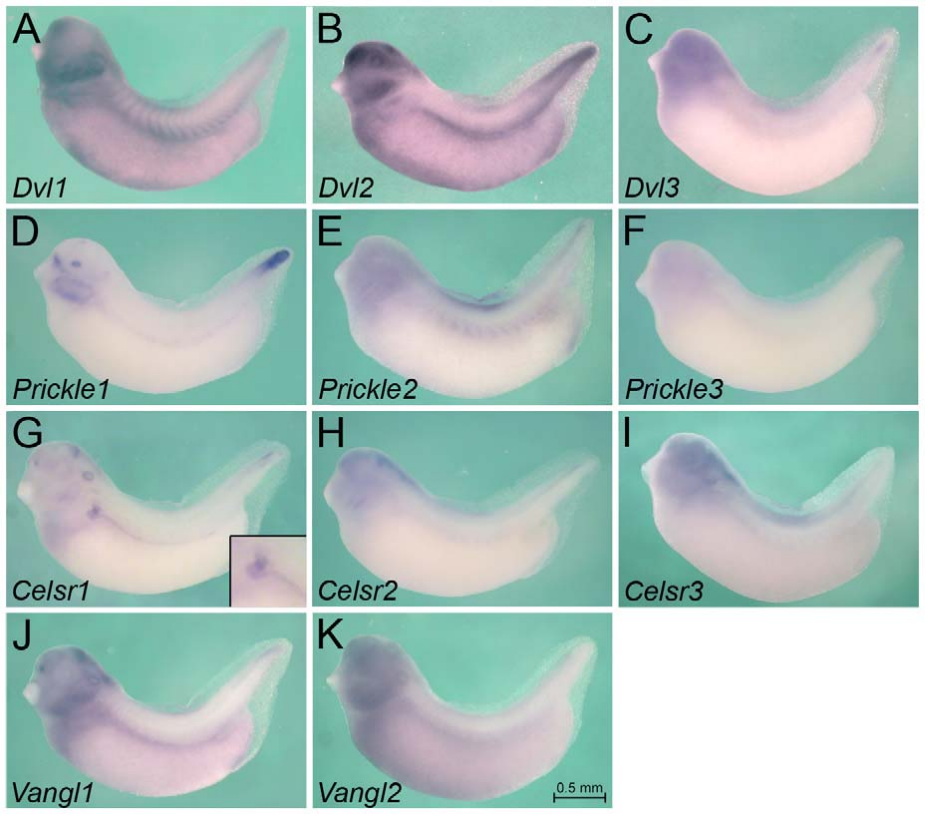
Expression of Wnt Signaling Intermediates at Stage 35. Whole mount *in situ* hybridization of *Xenopus* embryos at stage 35 for *Dvl1* (A), *Dvl2* (B), *Dvl3* (C), *Prickle1* (D), *Prickle2* (E), *Prickle3* (F), *Celsr1* (G), *Celsr2* (H), *Celsr3* (I), *Vangl1* (J) and *Vangl2* (K). Inset in G shows close-up of the pronephric tubular region. All images are of the same magnification and the scale bar corresponds to 0.5 mm.

**Figure 7:**
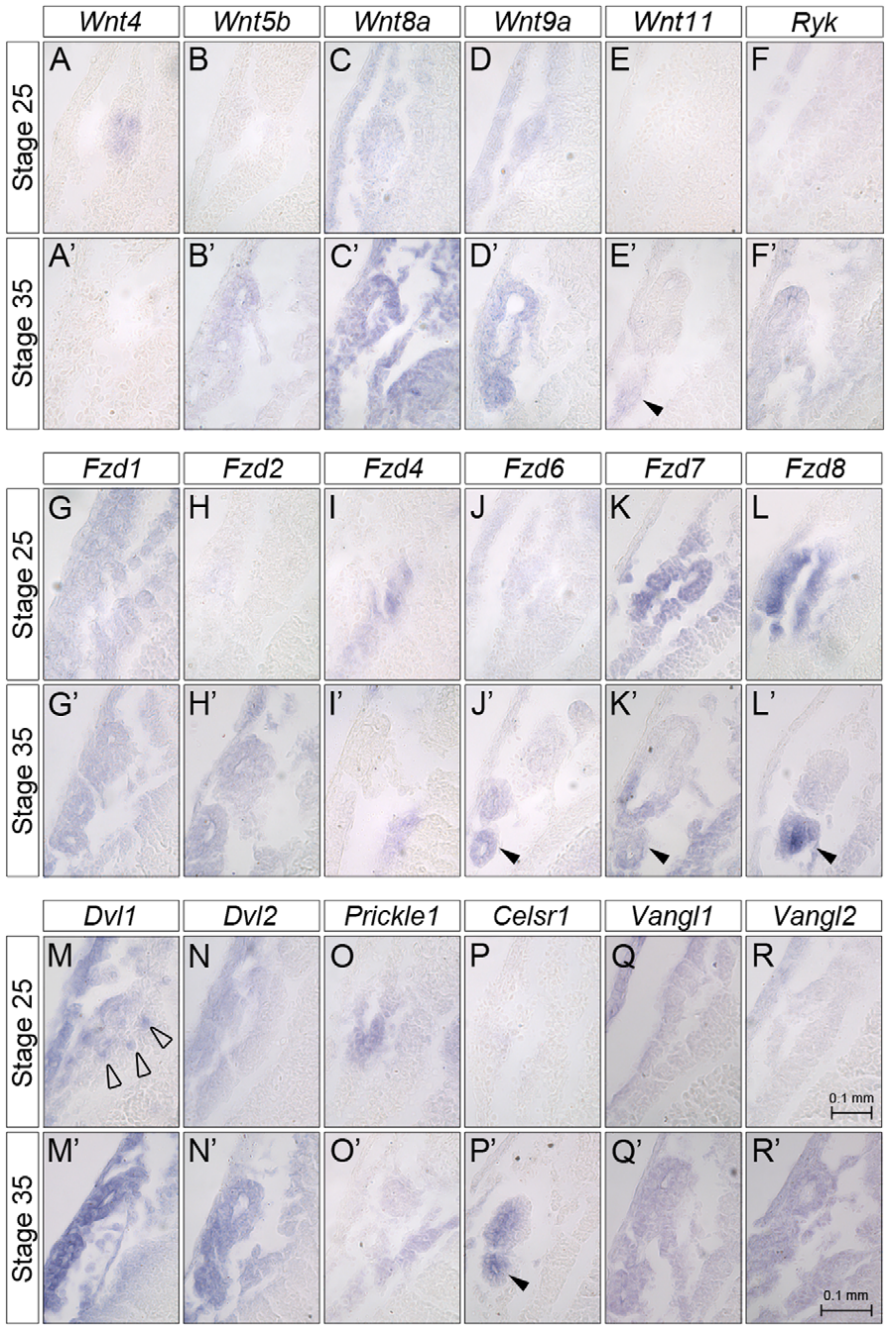
Pronephros Expression of Wnt Signaling Components. Paraplast sections of *Xenopus* embryos processed for whole mount *in situ* hybridization with *Wnt4* (A,A’), *Wnt5b* (B,B’), *Wnt8a* (C,C’), *Wnt9a* (D,D’), *Wnt11* (E,E’), *Ryk* (F,F’), *Fzd1* (G,G’), *Fzd2* (H,H’), *Fzd4* (I,I’), *Fzd6* (J,J’), *Fzd7* (K,K’), *Fzd8* (L,L’), *Dvl1* (M,M’), *Dvl2* (N,N’), *Prickle1* (O,O’), *Celsr1* (P,P’), *Vangl1* (Q,Q’) and *Vangl2* (R,R’) at stage 25 and 35. Sections are through the tubular area of the pronephros. Closed arrowheads indicate distal tubular segments; open arrowheads point towards individual *dvl1*-positive cells. All panels depicting embryos at stage 25 and 35, respectively are of the same magnification; a representative scale bar corresponding to 0.1 mm is shown in panels R,R’.

Interestingly, from all the Wnt molecules only a few show localized expression in the pronephros (Figures 1, 2 and 7A-E’). As previously reported [18], [19], *Wnt4* is specifically expressed in the undifferentiated kidney mesenchyme as early as stage 18 (Figures 1D, 7A and data not shown). Its expression becomes restricted towards the proximal tubules, is only detected in the nephrostomes (the ciliated funnel that connect the nephrocoelom to the pronephric tubules) at stage 35 and disappears by stage 39 (Figure 2D and data not shown). In contrast to *Wnt4* mRNA, *Wnt9a* is only weakly expressed in the undifferentiated pronephric mesenchyme, but is upregulated upon epithelial differentiation (Figures 1M, 2M and 7D,D’). It is present in all segments of the pronephric tubules and the duct, but is absent from the glomus. Finally, Wnt11, which was previously known as Wnt11r [20] is not expressed in the pronephros at early stages, but can be weakly detected in the pronephric duct by stage 30 and is still present at stage 39 (Figures 1Q, 2Q and 7E, E’ and data not shown). Besides these Wnt molecules that show distinct expression in the pronephros two others, *Wnt5b* and *Wnt8a*, are more broadly expressed in the intermediate mesoderm including the kidney (Figures 1F,K, 2F,K and 7B-C’). Interestingly, their role in kidney development is still completely unclear and has not yet been investigated in any organism.

All other Wnt molecules did not show noteworthy expression in the pronephros. As expected Wnts were detected in additional regions (Figures 1 and 2) with some of them reported earlier [18], [19], [20], [21], [22], [23], [24], [25], [26], [27], [28], [29], [30]: *Wnt1*, *Wnt2b*, *Wnt3a*, *Wnt8b*, *Wnt10a* and *Wnt10b* are in the nervous system; *Wnt5a*, *Wnt5b*, *Wnt7a*, *Wnt9a*, *Wnt11*, *Wnt11b* and *Wnt16* are in the heart region; *Wnt3a*, *Wnt7c*, *Wnt8a*, *Wnt8b*, *Wnt9a*, *Wnt9b* and *Wnt10b* are in the epidermis; *Wnt7a* and *Wnt7b* are in the lateral plate mesoderm; *Wnt11* and *Wnt11b* are in the somites; finally, *Wnt3a* and *Wnt6* are in the ear.

Together these data support the notion that Wnt molecules are important for pronephric kidney development and probably regulate multiple aspects in its formation.

### Expression of Wnt Receptors

Next, we analyzed the Wnt receptors. Like most other higher vertebrates *Xenopus* has 10 Frizzled receptors (Fzd) and two non-Frizzled receptors, Ror2 and Ryk (Figure S2). All Frizzled receptors could be detected by *in situ* hybridization at the stages analyzed and several exhibited a distinct kidney expression. As previously reported [31], *Fzd8* is expressed in the pronephros anlage as early as stage 20 (Figures 3H, 7L and data not shown). Interestingly, upon tubular differentiation *Fzd8* mRNA expression shifts from the mesenchyme to the epithelium (Figures 4H and 7L’). It also becomes regionally restricted and can now be only detected in the distal tubule and the pronephric duct, but not in the proximal tubules or the glomus. A similar pattern is observed for *Fzd7*, which is also expressed initially in the pronephros anlage and then subsequently in the distal tubule and duct (Figures 3G, 4G, 7K,K’ and [32]). However, while the early expression of *Fzd7* is of similar intensity as *Fzd8,* its later expression appears weaker. A third Frizzled receptor, *Fzd6*, also displays the late epithelial expression in the distal tubule and duct very similar to *Fzd7* and *Fzd8* (Figures 4F and 7J’). However, *Fzd6* cannot be detected early on in the undifferentiated pronephric mesenchyme (Figures 3F and 7J).

*Fzd4* mRNA is present in the developing glomus (Figures 3D, 4D and 7I,I’). *Fzd1* expression is more broadly expressed at stages 25 and 35 and is not restricted to the pronephric kidney (Figures 3A, 4A and 7G,G’). Interestingly, it shows elevated expression in the nephrostomes (see inset in Figure 4A). *Fzd2* is not present in the pronephros at early stages, but can be detected in the proximal tubules by stage 35 (Figures 3B, 4B and 7H,H’). Finally, *Ror2* and *Ryk* mRNA show distinct expression domains during early embryonic development, but are rather ubiquitous including in the pronephros at stages 25 to 35 (Figures 3K,L, 4K,L, 7F,F’ and [33], [34]).

As expected [31], [35], [36], [37], [38], [39], [40], [41], [42], Frizzled receptors can be found in many other expression domains (Figures 3, 4, and 7): *Fzd1*, *Fzd2*, *Fzd3*, *Fzd6*, *Fzd7*, *Fzd9* and *Fzd10* are present in different aspects of the nervous system; *Fzd4* in the developing nasal placode; *Fzd5* and *Fzd7* in the eye; *Fzd7* and *Fzd8* in the heart; *Fzd6* in the somites and *Fzd2* in the hypaxial muscles; *Fzd2, Fzd6* and *Fzd10* in the presomitic mesoderm and the tip of the tail.

Surprisingly these data demonstrate that many Fzd receptors exhibit strong kidney-specific expression patterns. This suggests that not only the Wnt ligands, but also of their receptors are tightly controlled during development.

### Expression of Wnt Signaling Intermediates

As a final step to better understand Wnt signaling in the pronephric kidney, we next focused our attention on downstream components of the pathway. Dishevelled (Dvl) is the key element shared by canonical and non-conical Wnt signaling [43]. Most vertebrates have three Dvl family members, Dvl1, Dvl2 and Dvl3 (Figure S3A). The early embryonic expression of these three genes in *Xenopus* has been previously examined [44]. We could confirm their early expression patterns, even though we observed stronger staining in the epidermis (Figures 5A-C and 7M,N). At stage 35, *Dvl1* and *Dvl2* continue to be expressed in the nervous system, the skin, the somites, while *Dvl3* is mainly found in the head region and the tip of the tail (Figure 6A-C). Moreover, *Dvl3* staining was much less intense than the two other genes. In respect to the pronephric kidney, only *Dvl1* and *Dvl2* were detected. The expression was not restricted to the mesenchyme or the epithelial structures, nor did it show any spatial pattern (Figures 5A,B, 6A,B and 7M-N’). Curiously, *Dvl1* expression at stage 25 was not detected homogenously in the pronephric mesenchyme, but instead labeled scattered individual cells (indicated by open arrowhead in Figure 7M).

Next, we explored the PCP signaling components that have been identified in *Xenopus* (Figure S3B-D). *Vangl1* and *Vangl2* mRNA expression is very similar to the one of *Dvl1* or *Dvl2*; both genes are present in the pronephros, but do not show any distinct patterns (Figures 5J,K, 6J,K and 7Q-R’). Conversely, the *Prickle* and *Celsr* families show more distinct patterns. *Prickle1* is expressed in the pronephric mesenchyme at stage 25 (Figures 5D and 7O), is strongly reduced upon tubulogenesis, but remains present in the mesenchyme adjacent to the tubular structures (Figures 6D and 7O’). In agreement with a previous report [45] *Prickle1* is also present in the presomitic mesenchyme, the tailbud, the eye lens and neural crest derivatives. In contrary to *Prickle1*, the other two family members, *Prickle2* and *Prickle3*, are not present in the kidney. *Prickle2* is found diffusely in the head region and the somites, while *Prickle3* is only very weakly detected in the brain (Figures 5E,F and 6E,F).

In respect to the Celsr family, no family member is expressed early on in the kidney, but at stage 35 *Celsr1* is specifically expressed in the distal tubule and pronephric duct (Figures 5G-I, 6G-I and 7P,P’). It is also detected in the ear, the brain, the posterior end of the neural tube and the heart. *Celsr2* and *Celsr3* are mainly present in the head region with *Celsr2* showing distinct labeling of the developing eye at stage 25.

Taken together these expression patterns demonstrate that some Wnt signaling components (i.e. *Dvl* and *Vangl*) are rather uniformly expressed, while others (i.e. *Prickle* and *Celsr*) are more regionally restricted.

## Discussion

**Figure 8:**
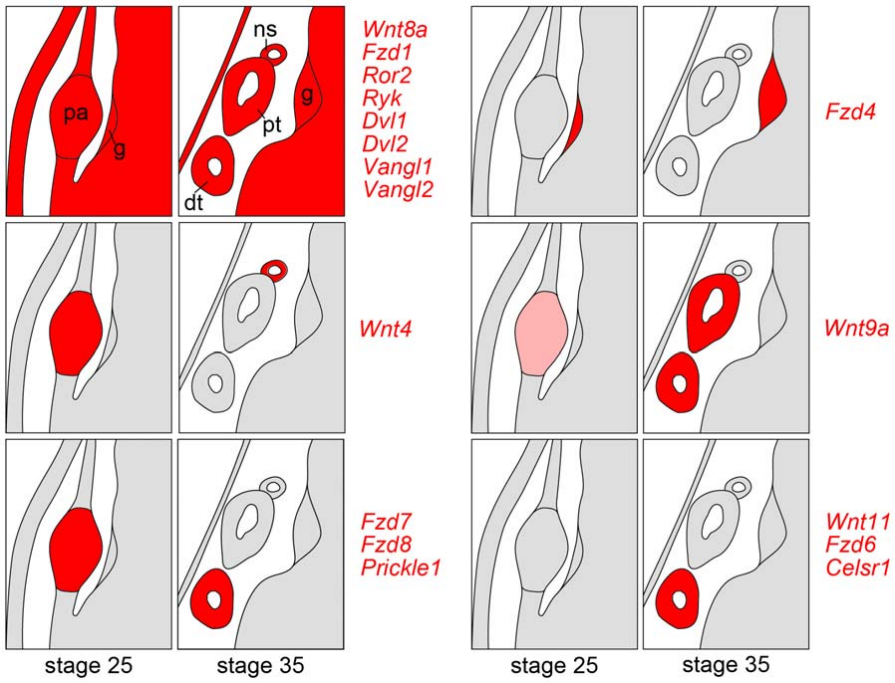
Summary of Different Wnt Signaling Expression Patterns. Schematics of stage 25 and 35 cross sections summarizing the *in situ* hybridization data; genes following the same expression pattern are grouped together and their respective expression domains are indicated in red (or light red in the case of weak expression). dt; distal tubules; g, glomus; pa, pronephric anlage; pt, proximal tubules; ns, nephrostomes.

Wnt signaling has been shown to be instrumental in the development of the pronephric and metanephric kidney at multiple individual steps [7], [46], [47]. Here we explored the entire signaling network by examining the expression of many of its components in *Xenopus laevis*. This analysis re-confirmed previous observations, but also discovered new areas of Wnt signaling that have not yet been described (Figure 8). They support the hypothesis that pronephros development can be subdivided into two distinct processes, the specification of the early pronephric mesenchyme and the late tubulogenesis events [47].

### Canonical Wnt signaling in the specification of renal progenitor cells

In the mouse, early nephron development is regulated by the sequential function of two Wnt ligands, Wnt9b and Wnt4. While Wnt9b regulates the self-renewal and differentiation of the mesenchymal nephron progenitors [13], [14], Wnt4 regulates the transition of pre-tubular aggregates into renal vesicles [15], [16]. An equivalent to the latter function has been described in *Xenopus*, where *Wnt4* is expressed during early pronephros development and is required for the formation of the proximal tubules [19]. However, *Wnt9b* is not found in the kidney at any stage and is restricted to the epidermis (Figures 1N and 2N and [22]). Interestingly, the *Wnt9b* paralogue, *Wnt9a*, is robustly expressed later in the kidney once epithelialization has occurred. It, thus, may have an equivalent function as mouse Wnt9b in tubulogenesis (see below), but does not share its role in nephron induction. This leaves the two possibilities that either the pronephric mesenchyme does not need to be maintained in a similar fashion as its metanephric counterpart or that another Wnt molecule takes its place. Indeed, *Wnt8a* is expressed throughout the mesoderm from gastrula stage onwards (data not shown and [28]) and is present at the right time and place to regulate the pronephros anlage. Such a function for Wnt8a in nephron development may have evaded detection since it is pivotal for early mesoderm formation in *Xenopus* [48], [49].

In respect to the Wnt receptors utilized in pronephros specification, several candidates, *Fzd1*, *Fzd4*, *Fzd7*, *Fzd8*, *Ror2* and *Ryk*, are expressed in the respective time period (Figures 3, 8 and data not shown). Two of them can be eliminated as likely targets: *Fzd4* is only localized to the glomerular domain and not the remainder of the kidney; Ror2 signals via Jnk and is β-Catenin-independent [50]. Since the early stages of pronephros development are thought to be β-Catenin-dependent [46] Ror2 is therefore an unlikely candidate. The contributions of the other receptors are more difficult to assess. They are probably - at least in part - redundant. Indeed, even though strongly expressed during the earliest phases of pronephros development, loss-of-Fzd8 or -Fzd7 does not interfere with the pronephros initiation, but rather with the later differentiation events ([31] and our own unpublished observations). Similarly, Ryk has been shown to not function alone but instead to be a Wnt co-receptor [51]. One revealing aspect is the syn-expression of *Fzd1* and *Wnt4* in the nephrostomes at stage 35 (Figures 2D and 4A) suggesting that Wnt4 signaling involves Fzd1.

### Non-canonical Wnt signaling in the morphogenesis of renal epithelial cells

Upon formation of the epithelial structures the observed expression patterns change probably reflecting a change from canonical to non-canonical Wnt signaling [47]. While *Wnt4* expression ceases and becomes restricted to the nephrostomes, other Wnts start being expressed. *Wnt9a* is strongly expressed in most segments of the pronephric tubules and duct and *Wnt11* is present in the pronephric duct only (Figures 2M,Q and 7D’,E’). Both of them have been implicated in regulating cell behavior in the mouse kidney. The Wnt9b paralogue, Wnt9a, regulates convergent-extension movements in proximal tubules and orientated cell division in the collecting ducts [52] and Wnt11 coordinates ureteric bud branching via a still poorly understood mechanism [53]. Similarly in *Xenopus*, the morphogenetic movements forming the highly conserved, three-dimensional tubular structure of the pronephros are regulated by non-canonical Wnt signaling [54]. However, the components of the Wnt signaling pathway involved in these processes are still unknown. While Wnt11 morphants exhibit a smaller pronephros, this effect is due to the expression of *Wnt11/11b* in the somites, but not the later expression in the pronephric duct [55]. In respect to the receptors, Fzd1, Fzd6, Fzd7, Fzd8, Ror2 and Ryk are candidates. Considering that Wnt9a and Wnt11 signal in a β-Catenin-independent fashion, Fzd6, Fzd7, Fzd8 and Ror2 are the more promising candidates. In particular, Fzd6 has been implicated in non-canonical Wnt signaling, since it regulates hair patterning and - in conjunction with Fzd3 - neural tube closure as well as hair bundle orientation of the inner ear [56], [57].

### Planar cell polarity signaling

As expected many downstream signaling components in particular the pan-Wnt signaling components *Dvl1* and *Dvl2*, or the PCP components *Vangl1* and *Vangl2* do not show any regionalization and instead are expressed continuously throughout pronephric kidney development. Surprisingly, two PCP signaling components, *Prickle1* and *Celsr1* did not follow this theme. In *Drosophila*, Prickle, Flamingo (the Celsr homologue), Vangl and Diego function together to regulate PCP in e.g. bristle orientation and ommatidial rotation [58]. However, in *Xenopus*, *Prickle1* or *Celsr1* mRNAs are detected in distinct and temporally non-overlapping expression domains in the kidney. While *Vangl1/2* are expressed throughout the developing pronephros and could presumably interact with both proteins, a complex of all three molecules is unlikely. Interestingly, the dichotomy in their expression patterns is not only observed in the pronephros, but also in the other expression domains (Figure 6D,G and [45]): *Prickle1* is present in the lens of the eye, while *Celsr1* is in the retina; *Prickle1* mRNA is restricted to the dorsal aspect of the ear, while *Celsr1* mRNA is found throughout; finally, *Celsr1* is present in the heart and *Prickle1* is absent. Moreover, the two other *Celsr* and *Prickle* molecules (*celsr2*, *celsr3*, *prickle2* and *prickle3*) exhibit diffuse and rather low expression. Together these expression data support the hypothesis that a classical PCP signaling pathway involving all core components is absent in the pronephros and probably in some of the other organ systems.

### Outlook

Our expression analysis supports the notion that pronephros development is regulated by an interplay between canonical and non-canonical Wnt signaling. Our study identified candidates for both processes. Interestingly, at either stage 25 or 35 multiple Wnt ligands, receptors and effector molecules are expressed. This suggests that a high degree of redundancy exists and that single loss-of-function studies may only reveal part of the whole process. Indeed, none of the loss-of-function experiments *in Xenopus* has so far resulted in embryos completely lacking a pronephros. Nevertheless, *Xenopus* is an ideal system to explore redundancy issues. As previously shown [59], [60], [61], injection of multiple antisense morpholino oligomer can target several genes at the same time. A similar approach in mouse requires the generation of double or triple knockouts, a process that is very time consuming. The multiple Wnt components identified here will provide a perfect starting point for this kind of analysis and hopefully will reveal novel aspects of kidney development.

## Materials and Methods

### Ethics Statement

This study was carried out in strict accordance with the recommendations in the Guide for the Care and Use of Laboratory Animals of the National Institutes of Health. The protocol was approved by the IACUC committee of the LSU Health Sciences Center (IACUC protocol #2760).

### *Xenopus* Embryos and *In Situ* Hybridization

*Xenopus* embryos obtained by *in vitro* fertilization were maintained in 0.1x modified Barth medium [62] and staged according to Nieuwkoop and Faber [63].

Whole mount *in situ* hybridizations and *in situ* hybridizations on paraplast sections were performed as described previously [64]. To generate antisense probes plasmids were linearized and transcribed as follows: *pBSK(-)-Wnt1*-*EcoRI*/T7 (NIBB #XL020a18), *pGEM-T-Easy-Wnt2b*-*NcoI*/Sp6, *pGEM-T-Easy-Wnt3a - SalI*/T7, pGEM2*-Wnt4*- *Nhe1*/T7 [23], *pGEM-T-Easy-Wnt5a*-*ApaI*/Sp6, *pCMV-SPORT6-Wnt5b - EcoRI*/T7 (IMAGE: 5048927), *pGEM-T-Easy-Wnt6*-*ApaI*/Sp6, *pGEM-T-Easy-Wnt7a*-*ApaI*/Sp6, *pCMV-SPORT6.1-Wnt7b*-*PstI*/T7 (IMAGE:7019251), *pCS111-Wnt7c - SmaI*/T7 (IMAGE: 8532605), *pCS2-Wnt8a*-*XhoI*/T3, *pBSKS-Wnt8b* - *NotI*/T7 (NIBB #XL040e10), *pBSKS-Wnt9a* - *ClaI*/T7, *pBSKS-Wnt9b* - *HindIII*/T7, pBSK(-)*-Wnt10a*-*EcoRI*/T7 (NIBB #XL100k05), *pGEM-T-Easy-Wnt10b - Nco1*/Sp6, *pBSK(-)-Wnt11* [20] - *NotI*/T7, *pBSK(-)-Wnt11b* - *EcoRI*/T7 (NIBB #XL092j06), *pGEM-T-Easy-Wnt16-NcoI*/T7 (GenBank Accession Number pending), *pExpress1-Fzd1-SmaI*/T7 (IMAGE: 7392077), *pCMV-SPORT6-Fzd2-EcoRI*/T7 (IMAGE: 3399141), *pBSK(-)-Fzd3*- *ClaI*/T7 (NIBB #XL260j03ex), *pBSK(-)-Fzd4*-*EcoRI*/T7 (NIBB #XL024e15), *pCMV-SPORT6-Fzd5- BamHI*/T7 (IMAGE: 3401522), *pBSK(-)-Fzd6*- *XbaI*/T7 (NIBB #XL220p06), *pCMV-SPORT6-Fzd7*-*EcoRI*/T7 (IMAGE: 5570875), *pBSKS-Fzd8*-*EcoRI*/T7, *pCS107-Fzd9- EcoRI*/T3 (IMAGE: 3200715), *pGEM-T-Easy-Fzd10-SalI*/T7, *pBSK(-)-Ror2*- *PstI*/T7 (NIBB #XL105o06), *pBSK(-)-Ryk* - *XbaI*/T7 (NIBB #XL049d20), *pCMV-SPORT6-Dvl1- Asp718*/T7 (IMAGE: 6956452), *pCMV-SPORT6-Dvl2- EcoRI*/T7 (IMAGE: 3401885), *pCMV-SPORT6-Dvl3- Asp718*/T7 (IMAGE: 6317854), pBSK(-)*-Prickle1* - *BamHI*/T7 (NIBB #XL141p03), *pGEM-T-Easy-Prickle2- SalI*/T7, *pCS111-Prickle3-BamHI*/T7 (IMAGE: 8824324), *pBSK(-)-Celsr1*-*EcoRI*/T7 (NIBB #XL005e01), *pCS105-Celsr2*-*EcoRI*/T3 (NIBB #XL513h08ex), *pGEM-T-Easy-Celsr3- NcoI*/Sp6, *pBSK(-)-Vangl1*-*PstI*/T7 (NIBB #XL038m21), and *pCMV-SPORT6-Vangl2-EcoRI*/T7 (IMAGE: 7010992). The identity of the individual family members was confirmed by multi-sequence alignment with the human, mouse, chick and zebrafish homologues using ClustalW2 or MUSCLE and visualized using the Phylogeny.fr web service [65]. In individual cases the alignment was confirmed by the inspection of the synteny.

## Supporting Information

Figure S1 **Phylogenetic Tree of Wnt Ligands.** Analysis of all Wnt ligands from human, mouse, chick, zebrafish and *Xenopus* using the MUSCLE algorithm.
(PDF)

Figure S2 **Phylogenetic Tree of Frizzled Receptors.** Analysis of all Frizzled receptors from human, mouse, chick, zebrafish and *Xenopus* using the ClustalW2 algorithm.
(PDF)

Figure S3 **Phylogenetic Tree of Wnt Signaling Intermediates.** Analysis of all disheveled (A), prickle (B), celsr (C) and vangl proteins (D) from human, mouse, chick, zebrafish and *Xenopus* using the ClustalW2 (C) or MUSCLE (A, B, D) algorithm.
(PDF)
