# Supplementary material for: Expression of Wnt Signaling Components during Xenopus Pronephros Development
Source: PLoS One. 2011 Oct 19;6(10):e26533. doi: 10.1371/journal.pone.0026533 (PMC3197532; doi:10.1371/journal.pone.0026533)
Supplement: Figure S2 — Phylogenetic Tree of Frizzled Receptors. Analysis of all Frizzled receptors from human, mouse, chick, zebrafish and Xenopus using the ClustalW2 algorithm. (PDF) [file pone.0026533.s002.pdf]

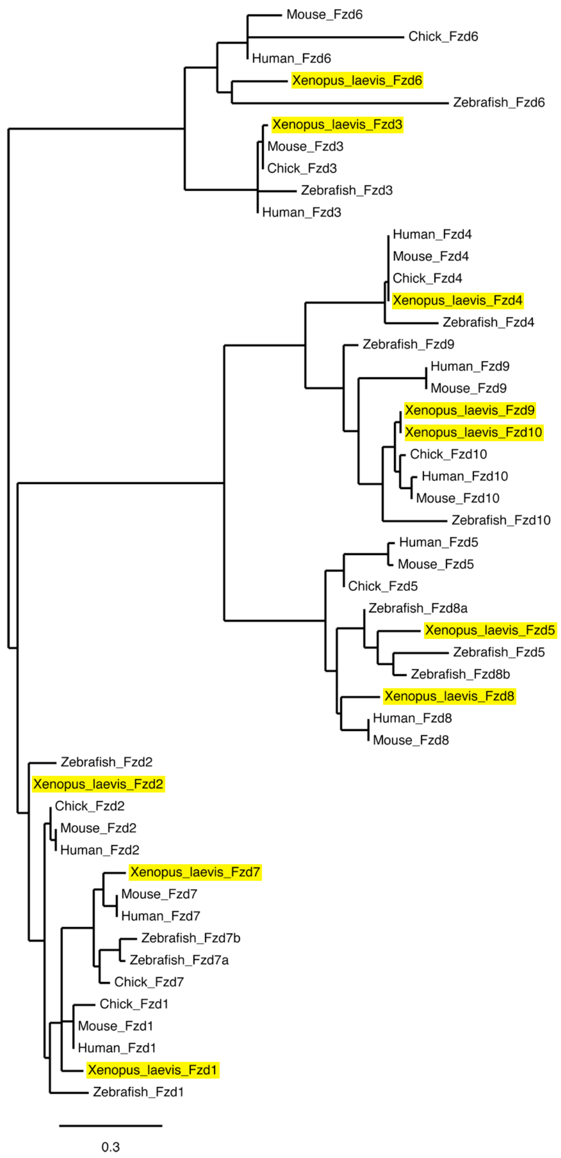

Supporting Figure S2. Phylogenetic Tree of Frizzled Receptors. Analysis of all Frizzled receptors from human, mouse, chick, zebrafish and *Xenopus* using the ClustalW2 algorithm.
