# Supplementary material for: Expression of Wnt Signaling Components during Xenopus Pronephros Development
Source: PLoS One. 2011 Oct 19;6(10):e26533. doi: 10.1371/journal.pone.0026533 (PMC3197532; doi:10.1371/journal.pone.0026533)
Supplement: Figure S3 — Phylogenetic Tree of Wnt Signaling Intermediates. Analysis of all disheveled (A), prickle (B), celsr (C) and vangl proteins (D) from human, mouse, chick, zebrafish and Xenopus using the ClustalW2 (C) or MUSCLE (A, B, D) algorithm. (PDF) [file pone.0026533.s003.pdf]

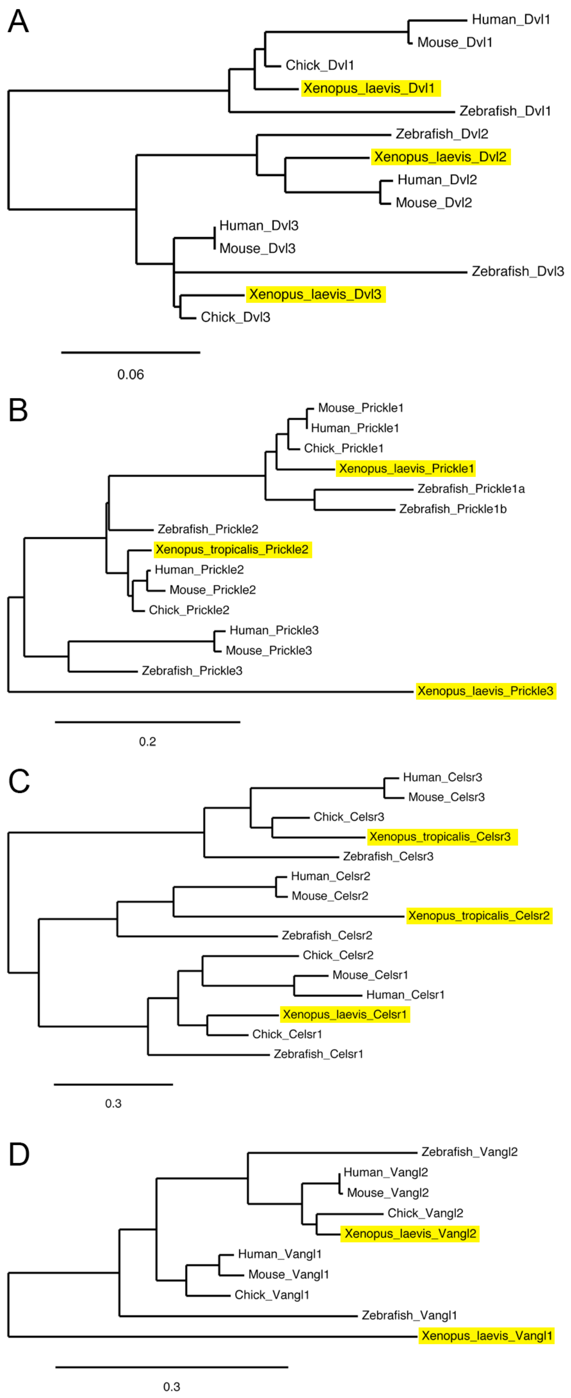

Supporting Figure S3. Phylogenetic Tree of Wnt Signaling Intermediates. Analysis of all disheveled (A), prickle (B), celsr (C) and vangl proteins (D) from human, mouse, chick, zebrafish and *Xenopus* using the ClustalW2 (C) or MUSCLE (A, B, D) algorithm.
